# Two-year minimum survivorship and radiographic analysis of a pressfit short humeral stem for total shoulder arthroplasty

**DOI:** 10.1016/j.jseint.2023.10.011

**Published:** 2023-11-22

**Authors:** Gabriel Larose, William R. Aibinder, Alexander T. Greene, Christopher P. Roche, Sean Grey, Kenneth J. Faber, Howard Routman, Samuel Antuña, Thomas Wright, Pierre-Henri Flurin, Joseph D. Zuckerman, Mandeep S. Virk

**Affiliations:** a Department of Orthopedic Surgery, NYU Langone Orthopedic Hospital, New York, NY, USA; b Department of Orthopaedic Surgery, University of Michigan, Ann Arbor, MI, USA; c Exactech, Inc., Gainesville, FL, USA; d Orthopedic and Spine Center of The Rockies, Fort Collins, CO, USA; e Roth|McFarlane Hand and Upper Limb Centre, London, Canada; f Palm Beach Shoulder Service at HCA Atlantis Orthopedics, Palm Beach, FL, USA; g Universidad Autónoma, Madrid, Spain; h University of Florida, Gainesville, FL, USA; i Clinique du Sport, Bordeaux, France

**Keywords:** Total shoulder arthroplasty, Short stem, Stress shielding, Canal filling, Radiographic outcomes

## Abstract

**Background:**

Newer generation humeral stem designs in total shoulder arthroplasty (TSA) are trending towards shorter lengths and uncemented fixation. The goal of this study is to report a 2-yr minimum clinical and radiographic outcomes of an uncemented short-stem press-fit humeral stem in anatomic total shoulder arthroplasty (ATSA) and reverse total shoulder arthroplasty (RTSA).

**Methods:**

A retrospective multicenter database review was performed of all patients who received an uncemented short-length press-fit humeral stem (Equinoxe Preserve humeral stem, Exactech, Inc., Gainesville, FL, USA) in ATSA and RTSA with a minimum two-year follow-up. The primary outcome was the prevalence of humeral stems at risk of radiographic loosening. Secondary outcomes included evaluation of functional outcome scores and prevalence of revision TSA for humeral stem loosening. Two blinded observers performed radiographic analyses, which included humeral stem alignment, canal filling ratio, radiolucent lines, stress shielding (calcar and greater tuberosity), and changes in component position (subsidence and stem shift). At risk stems were defined by the presence of one or more of the following: humeral stem with shifting or subsidence, scalloping of the humeral cortex, or radiolucent lines measuring 2 mm or greater in 3 or more zones.

**Results:**

287 patients (97 ATSA and 190 RTSA) were included in this study. The mean follow-up was 35.9 (±6.1) months. There were significant improvements for all functional outcome scores (*P* < .05), range of motion (*P* < .05), and visual analogue pain scale pain (*P* < .05). The prevalence of humeral stem at risk of radiographic loosening was 1% in the ATSA group (1/97) and 18.4% in the RTSA group (35/190). Calcar resorption was seen in 34% of ATSA and 19% of RTSA, with severe resorption in 12.4% of ATSA and only 3.2% of RTSA. Greater tuberosity resorption was present in 3.1% of ATSA and 7.9% of RTSA. The mean canal filling ratio was 50.2% (standard deviation 11.2%). Using logistic regression, a significant positive correlation between canal filling ratio and stress shielding (*P* < .01) was seen for both calcar and tuberosity stress shielding. The revision surgery rate was 0% in ATSA compared to 1.6% in RTSA.

**Conclusion:**

This retrospective study demonstrates a low revision rate and low prevalence of humeral stems at risk of radiographic loosening at two years with a press-fit short-stem humeral design in ATSA. Physiologic subsidence of humeral stems can account for higher prevalence of humeral stems at radiographic risk of loosening in RTSA compared to ATSA

Total shoulder arthroplasty has been shown to have good outcomes in treating end-stage shoulder pathologies.^28^ Historically, humeral stems were cemented. The cemented stems have been shown to have a low loosening and subsidence rate,^26^^,^^35^ with a rate of humeral stem loosening of 1.4% in a recent meta-analysis.^33^ In the same analysis, the revision rate of the cemented humeral stem is reported at 2.3% at 29 months.

Modifications in the press-fit humeral stems including shorter stem length and metaphyseal fixation have generated increasing interest in this stem design, with more and more surgeons using them.^20^ The benefits of a press-fit stem includes decreased operative times and simplified revision with bone preservation.^15^^,^^20^ Standard-length press-fit stems now have a long track record with a low rate of loosening.^5^^,^^16^^,^^21^^,^^31^^,^^37^ A recent meta-analysis showed a pooled loosening rate of 3.8%^33^ and a low revision rate (1.8% at 35 months^33^). However, standard-length stems (>90 mm) violate the medullary canal and create a risk for intraoperative complications such as periprosthetic humerus fractures, which are reported to be between 1.5%-16%.^10^ Additionally, standard length press-fit humeral have been associated with a high rate of proximal stress shielding with complete or partial resorption of the greater tuberosity and calcar as high as 100% and 76%, respectively.^17^

Metaphyseal fit short humeral stems (70-90 mm) were designed to be a load-sharing stem, with the theoretical goal of minimizing proximal stress shielding.^12^ By only impacting the metaphysis with a broach, they allow for preserving bone, ease of revision, and less stress riser in the diaphysis.^12^ However, recent studies illustrated a higher rate of stem loosening up to 8%.^2^^,^^4^^,^^8^^,^^9^^,^^19^^,^^25^^,^^27^ Furthermore, these studies have demonstrated variable rates of calcar and greater tuberosity stress shielding.^2^^,^^7^^,^^22^ These studies included different stem designs, coatings and follow-up timepoints. Stem design may play a role in stress shielding, bony adaptations and implant survivorship.

The goal of this study was to review the short-term radiographic and clinical outcomes of a short-stem press-fit humeral design in anatomic total shoulder arthroplasty (ATSA) and reverse total shoulder arthroplasty (RTSA) at a minimum of 2 years. We hypothesize that this short stem will have a good clinical outcome, a low incidence of stem at risk of loosening, and low stress shielding with low bone resorption.

## Method

### Study design: retrospective study

#### Patient cohort

This is a multicenter, retrospective study of patients undergoing ATSA or RTSA using a short stem, press fit humeral implant (Exactech Preserve stem, Exactech, Inc., Gainesville, FL, USA). To be included in the study patients had to have a minimum of 2 years clinical and radiographic follow-up after a primary ATSA or RTSA performed by one of 8 upper extremity surgeons. Exclusion criteria included acute proximal humerus fracture, revision shoulder arthroplasty, hemiarthroplasty, and cemented humeral stems; a research ethic board review was obtained from each center for this study.

#### Implant’s description

**Fig. 1:**
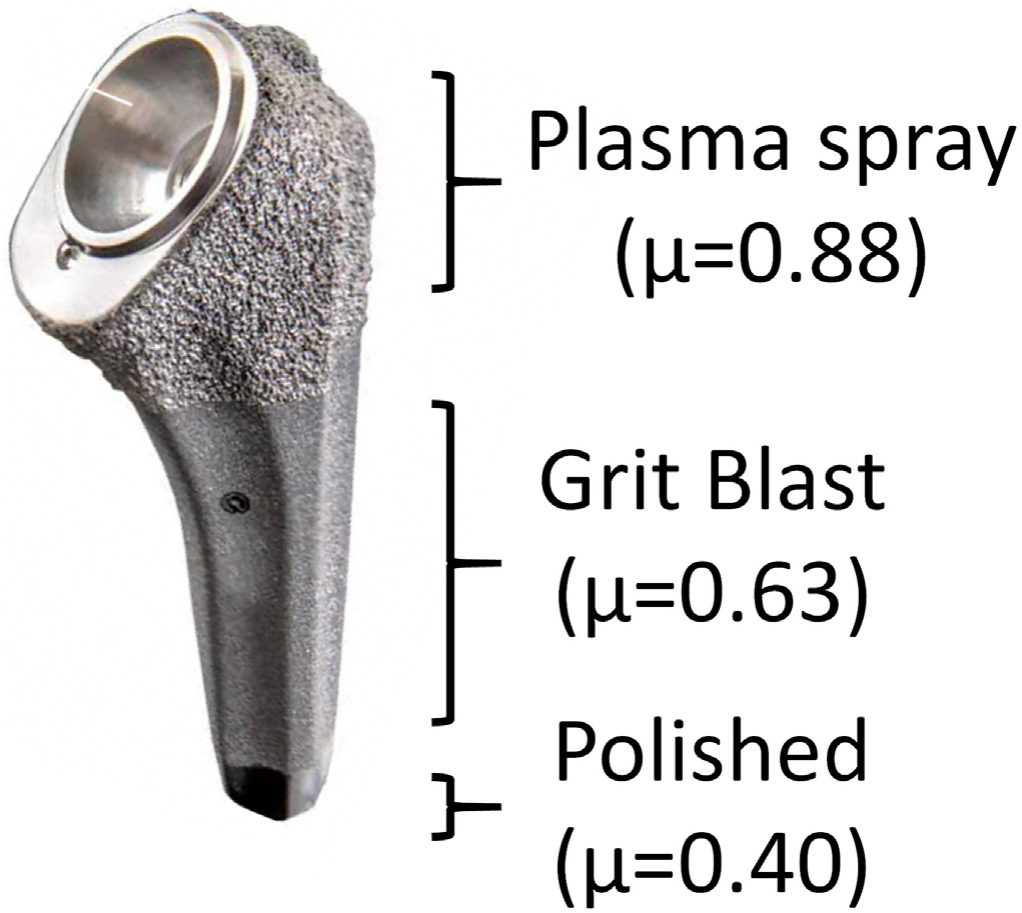
Anatomy of the short stem (μ = Micron).

The humeral stem is a convertible onlay implant with a tapered stem design and a larger proximal body for metaphyseal fixation. The proximal aspect of the implant is plasma sprayed for bony ingrowth and the rest of the implant is grit blasted (Fig. 1). The implant has a fluted geometry proximally to improve the torsional stability and increase the contact surface area in metaphyseal bone. For ATSA applications, the 1.5 mm and 4.5 mm offset replicator plates and the 1.5 mm offset humeral heads permit independent adjustment of humeral head offset, inclination and version to reconstruct the native humeral anatomy. A fixed angle metaphyseal tray is used for RTSA reconstruction

#### Surgical technique

All surgeries were performed via a standard deltopectoral approach in beach chair position. The same humeral stem preparation technique was used for ATSA and RTSA. After making the humeral head cut, a 6 mm entry reamer was used to access the humeral canal. No further canal reaming is necessary and sequentially broaching of the proximal metaphysis is performed to find the interference fit. Broaching with this humeral stem design involves metaphyseal impaction. Version during broaching for ATSA and RTSA was as per surgeon’s preference. The humeral component size is 0.5 mm larger than the size of the corresponding broach to allow for press fit fixation with good torsional stability. The size of the stem was clinically decided at the time of broaching to obtain a stable press-fit. The platform stem design allows for convertibility between ATSA and RTSA. In ATSA, the subscapularis was addressed with either a tenotomy, a peel, or an osteotomy and repaired at the end with nonabsorbable sutures. In RTSA, subscapularis was repaired if present at the end of the case or not repaired at all depending on surgeon’s preference. Glenoid preparation was then done depending on the implant design (ATSA or RTSA) selected.

#### Outcome measures

The primary outcome was the prevalence of humeral stems at risk of radiographic loosening following the definition of Sperling et al^29^ with the presence of one of the following: evidence of subsidence on the radiographs, evidence of tilt of the implant, or presence of radiolucency of more than 2 mm in 3 zones or more. Secondary outcomes included evaluation of functional outcome scores and prevalence of revision TSA for humeral stem loosening.

Deidentified radiographs (immediately postoperative and 2 year postoperative) were reviewed by two blinded shoulder and elbow fellowship trained observers (GL & WRA). The mean of both values was used to analyze the outcomes of the study. This included determination of humeral stem alignment in the canal and canal filling ratio, the presence of radiolucent lines and stress shielding (calcar and greater tuberosity), and changes in component position (subsidence and change in alignment). Neutral alignment was defined as less than 5 degrees of varus or valgus angulation based on prior studies^2^^,^^9^^,^^13^^,^^22^ Stress shielding was graded as absent, incomplete (decreased bone mineral density) or complete (resorption of cancellous and cortical bone). Humeral stems at risk of radiographic loosening were determined by the aforementioned criteria.^29^

Secondary outcomes include patient-reported outcomes measures (PROMs): American Shoulder and Elbow Surgeons (ASES) score, Constant score, Simple Shoulder test, University of California-Los Angeles shoulder score, Shoulder Pain and Disability Index score, Shoulder Arthroplasty Smart score,^24^ and visual analogue pain scale (VAS). Shoulder range of motion (ROM) was recorded presurgery as well as 2 years postsurgery.

#### Statistical analysis

Descriptive statistics was used to report the radiographic outcomes. A Paired T-test was used to compare preoperative and postoperative PROMs and ROM. A logistic regression using the presence of bone resorption as the dependent variable and canal filling ratio as the independent variable was used to evaluate the correlation between them.

## Results

### Demographics and cohort characteristics

A total of 287 patients (97 ATSA and 190 RTSA) were recruited for this study. The minimum follow-up was two years (mean follow-up 35.9 ± 6.1 months). Patients’ demographics are shown in Table I. In the ATSA group, the main preoperative diagnosis was glenohumeral osteoarthritis or post-traumatic arthritis. In the RTSA, the main diagnoses were rotator cuff tear arthropathy and glenohumeral osteoarthritis (Table I).

**Table I:** Demographics.

	ATSA		RTSA		*P* value
	n	97	n	190	
	Mean	SD	Mean	SD	
Age (range)	65.0	(48-80)	69.6	(47-83)	<.05∗
Sex, female (%)	35	36.1%	99	52.1%	
Follow-up	35.8	5.2	35.9	56.5	.94
BMI	30.3	5.7	30.3	6.0	.95
Stem size (most frequent)	10.0		6.0		
Preoperative diagnosis					
Osteoarthritis	77		73		<.05∗
Rotator cuff arthropathy	0		61		
Post-traumatic arthritis	2		0		
No information	18		56		
Comorbidities					
none	33		41		.59
Inflammatory arthritis	5		15		
Hypertension	48		114		
Heart disease	11		50		
Diabetes	11		40		
Other	17		30		

*SD*, standard deviation; *BMI*, body mass index; *ATSA*, anatomic total shoulder arthropathy; *RTSA*, reverse total shoulder arthroplasty.

Other (includes Malignancies, Chronic Obstructive Pulmonary Diseases, Hypothyrodism, Multiple Sclerosis, Parkinsons, Liver Disease).

∗ P value <.05 was considered significant.

### Radiographic assessment

In the RTSA group, there were 35 (18.4%) at risk humeral stems (2 stems with lucencies of >2 mm in 3 zones; 8 stems with both implant subsidence and a change in alignment; 1 stem with lucencies, subsidence and a change in alignment; 21 stems with subsidence but no change in alignment; 3 stems with an implant change in alignment) (Table II). There was one stem at risk in the ATSA group. In the RTSA group, 81.1% had no calcar resorption, 15.8% had incomplete calcar resorption, and 3.2% had complete resorption. Tuberosity resorption was present in 7.9% of RTSA. In the ATSA, 66.0% had no resorption in the calcar, 21.6% had incomplete resorption, and 12.4% had completed. Tuberosity resorption was present in 3.1% of ATSA.

**Table II:** Radiographic outcomes.

	ATSA		RTSA	
	n	97	n	190
Stress shielding calcar				
None	64	66.0%	154	81.1%
Incomplete	21	21.6%	30	15.8%
Complete	12	12.4%	6	3.2%
Stress shielding tuberosity				
None	94	96.9%	175	92.1%
Present	3	3.1%	15	7.9%
Stem at risk				
Yes	1	1.0%	35	18.4%
No	96	99.0%	155	81.6%
Canal filling	46%	0.115	52%	0.104
Radiolucent lines				
<1 mm	91	93.8%	172	90.5%
1-2 mm	5	5.2%	15	7.9%
>2 mm	1	1.0%	3	1.6%
Revisions	0	0.0%	3	1.6%

*ATSA*, anatomic total shoulder arthroplasty; *RTSA*, reverse total shoulder arthroplasty.

Canal filling was 52% (±10) in the RTSA group compared to 46% (±11) in the ATSA. In the ATSA group, 74 (76%) patients had their stems put in neutral alignment (0 ± 5°). In the RTSA group, 140 patients (74%) had a neutral alignment stem. There was no correlation between malalignment and canal filling (*P* = .77). There were no differences in functional outcomes between patients with a neutral stem or a malalignment stem (*P* = .58 in ATSA and *P* = .35 in RTSA). There was a significant increase in calcar resorption in patients with canal filling ratio of >0.6 (odds ratio [OR]: 2.1, 95% confidence interval 1.2-4.0, *P* = .02) (Table III). The impact of canal filling ratio on bone resorption of the tuberosity approached significance with OR: 2.7, confidence interval: 1.0-7.4, *P* = .059.

**Table III:** Canal filling ratio.

Canal filling ratio cut off	Stress shielding of:	Odds ratio	95% confidence interval	*P* value
0.6	Calcar	2.14	1.15-3.99	.02
0.6	Tuberosity	2.72	1.01-7.36	.06

### Revision surgery

There were three patients that underwent a revision in the RTSA group. One patient had developed aseptic loosening of his baseplate at 27 months after the primary surgery and was revised to a hemiarthroplasty. The second patient had a fall causing failure of his baseplate at 19 months and needed revision of the baseplate. Finally, the third patient had an aseptic loosening of humeral stem, needing only stem revision. No revisions were needed in the ATSA group.

### Clinical assessment

All PROMs were significantly improved at the two years follow-up compared to the preoperative follow-up for both ATSA and RTSA (Tables IV and V). In the anatomic group, the VAS score decreased from 6.2 ± 2.1 to 1.1 ± 1.7 (*P* < .05). Similarly, in the reverse group, the VAS decreased from 6.1 ± 2.2 to 1.0 ± 1.8 (*P* < .05). The Constant score in the anatomic group improved from 53.7 ± 15.4 to 77.6 ± 9.1 (*P* < .05), and in the reverse group from 41.4 ± 14.1 to 70.6 ± 14.2 (*P* < .05). The ASES score improved from 42.0 ± 15.8 to 88.3 ± 13.6 (*P* < .05) in the anatomic group and 38.4 ± 14.7 to 85.1 ± 16.4 (<.05).

**Table IV:** Clinical outcomes ATSA.

	Preoperative		Postoperative		*P* value
	Mean	SD	Mean	SD	
Pain daily score	6.2	2.1	1.1	1.7	<.05∗
Pain worst score	8.6	1.3	2.3	2.7	<.05∗
ASES	42.0	15.8	88.3	13.6	<.05∗
Constant	53.7	15.4	77.6	9.1	<.05∗
SST	5.7	2.8	11.1	1.4	<.05∗
UCLA	16.8	3.8	32.0	3.5	<.05∗
SPADI	70.4	23.2	13.2	15.7	<.05∗
SAS	52.6	10.5	80.6	9.0	<.05∗
Active FF	121	35	160	16	<.05∗
Active ABD	107	38	153	23	<.05∗
Active ER	34	18	51	16	<.05∗

*ATSA*, anatomic total shoulder arthroplasty; *ASES*, American Shoulder and Elbow Surgeons score; *SST*, simple shoulder test; *UCLA*, University of California-Los Angeles Shoulder Score; *SPADI*, shoulder pain and disability index; *SAS*, shoulder arthroplasty smart score; *SD*, standard deviation; *FF*, forward flexion; *ABD*, abduction; *ER*, external rotation.

∗ Statistically significative.

**Table V:** Clinical outcomes RTSA.

	Preoperative		Postoperative		*P* value
	Mean	SD	Mean	SD	
Pain daily score	6.1	2.2	1.0	1.8	<.05∗
Pain worst score	8.8	1.6	2.2	2.9	<.05∗
ASES	38.4	14.7	85.1	16.4	<.05∗
Constant	41.4	14.1	70.6	14.2	<.05∗
SST	4.7	2.5	10.1	2.3	<.05∗
UCLA	14.7	4.0	31.2	5.0	<.05∗
SPADI	78.8	19.6	20.4	22.7	<.05∗
SAS	48.9	12.5	75.6	22.7	<.05∗
Active FF	107	42	144	26	<.05∗
Active ABD	96	43	136	30	<.05∗
Active ER	28	23	40	15	<.05∗

*RTSA*, reverse total shoulder arthroplasty; *ASES*, American Shoulder and Elbow Surgeons score; *SST*, simple shoulder test; *UCLA*, University of California-Los Angeles Shoulder Score; *SPADI*, shoulder pain and disability index; *SAS*, shoulder arthroplasty smart score; *SD*, standard deviation; *FF*, forward flexion; *ABD*, abduction; *ER*, external rotation.

∗ Statistically significative.

The ROM was also significantly improved for both anatomic and reverse TSA after the surgery (Tables II and III). In the anatomic group, the active forward flexion improved from 122 ± 35 to 160 ± 16 (*P* < .05), the abduction improved from 107 ± 38 to 153 ± 23 (*P* < .05), the external rotation improved from 34 ± 18 to 51 ± 16 (*P* < .05). In the reverse group, the active forward flexion improved from 107 ± 42 to 144 ± 26 (*P* < .05), the abduction improved from 96 ± 43 to 136 ± 30 (*P* < .05), the external rotation improved from 28 ± 22 to 40 ± 15 (*P* < .05).

## Discussion

This retrospective study aimed at reviewing the short-term follow-up of a new humeral short stem and compares it to the previous literature using different humeral implant design. Our study demonstrates a low rate of revision (0% in the ATSA group and 1.6% in the RTSA group) with a press-fit short humeral length stem. Our data also shows a low rate of radiolucent lines. The metaphyseal press-fit fixation and load sharing translates into lower prevalence of proximal stress shielding at 2 years than standard length stem.^18^^,^^22^^,^^23^ Our data shows a significant improvement in functional outcomes at two years post-TSA in both anatomic and reverse TSA.

Despite good clinical outcomes, a relatively high rate of stem loosening and unacceptable rate of revision of the humeral stem was initially reported with some other designs of a short humeral stems.^4^^,^^19^ The development of humeral stem with ingrowth coating, that are similar to stem coating used in total hip arthroplasty was seen as a solution to improve the biological fixation of short humeral stem.^19^ Recent studies have showed an improvement in the fixation of short humeral stems with proximal porous coating. The rate of stem at risk of loosening using Sperling et al^29^ description was reported to be between 0%-14%.^2^^,^^8^^,^^9^^,^^19^^,^^25^^,^^27^^,^^34^ However, all these studies have a limited follow-up of about 2 years. It is known that the rate of humeral loosening increase with longer follow-up.^11^ Using the traditional definition of stem at risk,^29^ the rate of stem at risk was 1% in the ATSA group (1 patient with implant subsidence and implant that tilted in varus) in our study. However, in the RTSA group, the rate of stem at risk seems relatively high 18.4%. In this group there were 21 patients with implant subsidence but no tilt or significant radiolucent lines. Although we report that these patients are at radiographic risk of loosening, the stems show minimal subsidence and did not develop any changes in alignment, significant radiolucent lines or clinical symptoms. Therefore, the reported high rate of stem at risk in the RTSA group in our study is most likely a result of what we think as physiologic subsidence in RTSA. This has been reported in previous studies.^32^^,^^34^ A recent paper from Tross et al^32^ report a 11% subsidence of more than 5 mm measured on postoperative radiographs. However, in their study, that subsidence was not associated with decrease in functional outcomes (Constant score, SSV, ROM) compared to patient with implant without subsidence. The stem subsidence was not correlated to canal filling ratio. They did not report any revision for loose stem at their final follow-up of 18 months. In a study using radio stereometric analysis, Van de Kleut et al^34^ analyzed the translation of the short press fit humeral stem over time. They reported progressive inferior translation of the humeral stem over the 2 years post implantation. The stem translation stabilized around 12 months post implantation. In our cohort, the rate of stem subsidence in the RTSA group without associated radiolucency or varus/valgus tilt was 15%. Similar to Tross et al study,^32^ we have no stem revisions for loosening in this subset of patients with stem subsidence. Based on the findings of Van de Kleut et al and our study, the metaphyseal press fit short stem subside slightly, especially in RTSA, before bony ingrowth happens to ensure stem stability. Of the 35 humeral stems at-risk of radiographic loosening in our study, only 3 were noted to have any lucent lines. If we exclude the patients with only subsidence, the new rate of stem at risk would be 7.9% (15/190), which is comparable to Zmistowski et al^36^

We consider that early subsidence is not a sign of stem at risk, rather part of the normal settling of this new stem design that is purely metaphyseal and do not rely on any cortical fixation. Furthermore, in this particular implant, the onlay humeral tray often sits on metaphyseal bone only without support of the peripheral cortical bone. Aibinder et al^2^ have demonstrated that a larger humeral tray, relative to the humeral metaphysis, prevents early stem subsidence much like a humeral head component in ATSA. This could also explain why this phenomenon was not seen in ATSA in our cohort. Future studies are necessary to quantify the amount of humeral stem subsidence in RTSA as well as the timeline to stability to define what is “safe” subsidence vs. subsidence that predisposes to future stem loosening.

There is higher risk of malalignment with short humeral stems when compared to standard length stems because in long stem that rely on the proximal disphysis for stem positioning. In this study, the humeral stems were in a neutral alignment (0 ± 5°) in 76% in the ATSA group and 74% in the RTSA group. Lädermann et al^13^ report a risk of malalignment (>5°) of 47%. They correlated the malalignment with a decrease in the canal filling ratio. However, Erickson et al^9^ only reported a 5% malalignment and no correlation with the canal filling ratio. The risk of malalignment is quite variable between studies and range from 5%-47%.^2^^,^^9^^,^^22^ Our data did not show a correlation between the malalignment and the canal filling ratio. Moreover, similarly to what was shown by Denard et al,^7^ we did not measure any difference in PROMs between patient in neutral alignment and patient with a malalignment. Additional surveillance is necessary to determine the impact of stem alignment on mid-term and long-term clinical outcomes.

Standard length uncemented stem have been associated with significant proximal stress shielding. Raiss et al^22^ reported stress shielding in 63% of regular length stem. Melis et al^18^ event reported tuberosity resorption in 100% of uncemented stems. Razfar et al^23^ demonstrated with a finite element analysis that reducing stem length results in proximal cortical bone stresses that more closely approximate stresses observed in normal bone. Therefore, short stems were developed with the goal of improving the stress shielding in calcar and greater tuberosity. In our study, 34% of patient in ATSA group had partial resorption of their calcar and 12.4% had complete resorption. In the RTSA group, 19% of patient had partial calcar resorption and 3.2% of patients had complete resorption. Tuberosity resorption was seen in 3.1% of patient in the ATSA group and 7.9% of patients in the RTSA group. These values of calcar and tuberosity resorption are similar to what was reported in previous studies for different short stem design. Aibinder et al^2^ reported stress shielding of the tuberosity in 14% of their patient and calcar resorption on 23%. Denard et al^7^ report partial calcar resorption in 23% of patients with short stem; however, the resorption was not associated with any difference in patients functional outcomes. Erickson et al^9^ reported a low risk (calcar 2.2%, tuberosity 2.9%) of resorption in patients treated with a short stem, which they attributed to low canal filling ration. However, compared to the other studies, which had a minimum follow-up of one year, our radiographic results had a minimum 2-year outcome. Raiss et al^22^ reported that 17% of their patients had high bone adaptation changes. These patients had a higher canal filling ratio (>0.7) with a relative risk of 4.1. Similarly to Raiss et al,^22^ our study shows an increase in bone adaptation and resorption with an increase in canal filling ratio. However, in our study, a canal filling ratio of >0.6 was associated with significant bone changes. Our study only had 12 (4.2%) patients who had a canal filling ratio >0.7. Thus, even though the OR for greater tuberosity stress shielding was 4.7 for this subset, the low numbers of patients made this not statistically significant which is why we used 0.6 as a threshold. The OR in our study for a filling ratio of 0.6 threshold was nearly half of the relative risk in the prior study. A recent finite element analysis study reported that smaller short stems reproduce cortical and trabecular stresses closer to the normal bone than a larger stem.^14^

Good functional and clinical outcomes have been reported with the use of short stem.^1^, ^2^, ^3^, ^4^^,^^6^^,^^25^^,^^27^^,^^30^^,^^32^ Erikson et al^9^ compared the functional outcomes of a short stem with a standard length stem at one year, and demonstrated no differences with respect to ASES, WOOS, and SANE scores. Similarly, Denard et al^6^ reported no significant differences in the ROM, VAS, Simple Shoulder test, ASES, and SANE score between the standard length and short length humeral stems. Our data is similar to previous studies that report that press fit short humeral stem provide good clinical outcomes for both ATSA and RTSA.

This study has some limitations, First, this is a retrospective multicentric study although radiographic and functional data is collected prospectively. Second, multiple surgeons from different institutions and geographic distribution participated in the study; therefore, in theory, surgical techniques and postoperative protocols can differ, and can have an impact on some of the radiographic outcomes. However, we consider this limitation as the strength with respect to external validity of the results. Third, we did not have a comparison group in this study, which limits the conclusion of the improvement of the short stem compared to traditional stem. We used the literature to compare the two stem’s lengths. Finally, two-year follow-up is relatively short term for measuring the outcome of this stem; however, important information need to be obtain in this short term follow-up to understand the behavior of this short stem.

## Conclusion

This retrospective study is the largest one reviewing the short-term outcome of this short humeral stem. It demonstrates a low revision rate and low prevalence of humeral stems at risk of radiographic loosening at two years with a press fit short-stem humeral design in ATSA. Physiologic subsidence of humeral stems can account for higher prevalence of humeral stems at radiographic risk of loosening in RTSA compared to ATSA. Therefore, longer follow-up will be needed to determine if the theoretical advantages of the shorter length humeral stem outweigh the potential risks related to misalignment and subsidence relative to a longer humeral stem.

## Disclaimers:

Funding: No external funding was received for the study.

Conflicts of interest: GL: (Paid lecture – ENOVIS); WRA (Paid Consultant – EXACTECH Inc.); AG (Paid employee – EXACTECH Inc.); CPR (Paid Employee and Stock – EXACTECH Inc.); SG (Paid Consultant and Royalties – EXACTECH Inc.); KJF (Paid Consultant and Royalties – EXACTECH Inc.); HR (Paid Consultant, Royalties, Stock, Patents – EXACTECH Inc.); SA(Paid Consultant and Royalties – EXACTECH Inc.); TW(Royalties, Patents, Stock – EXACTECH Inc.); PHF (Paid Consultant Royalties, Stock, Patents – EXACTECH Inc.); JDZ (Royalties and Stock – EXACTECH Inc.); MSV (Paid Consultant – EXACTECH Inc.). The other authors, their immediate families, and any research foundations with which they are affiliated have not received any financial payments or other benefits from any commercial entity related to the subject of this article.
